# Interleukin-33 deficiency prevents biliary injuries and repairments caused by *Clonorchis sinensis* via restraining type 2 cytokines

**DOI:** 10.1186/s13071-022-05490-6

**Published:** 2022-10-22

**Authors:** Chao Yan, Na Xu, Man Liu, Zhihua Jiang, Jing Wu, Stephane Koda, Yu Chen, Beibei Zhang, Qian Yu, Yin-Hai Xu, Jian-Lin Wu, Kui-Yang Zheng

**Affiliations:** 1 Jiangsu Key Laboratory of Immunity and Metabolism, Department of Pathogenic Biology and Immunology, National Demonstration Center for Experimental Basic Medical Science Education, Xuzhou Medical University, Xuzhou, People’s Republic of China; 2 Wuming Hospital of Guangxi Medical University, Nanning, Guangxi Zhuang Autonomous Region People’s Republic of China; 3 Institute of Parasitic Disease Control and Prevention, Guangxi Key Laboratory for the Prevention and Control of Viral Hepatitis, Guangxi Zhuang Autonomous Region Center for Disease Control and Prevention, Nanning, China; 4 Hengzhou Center for Disease Control and Prevention, Nanning, China; 5 Department of Laboratory Medicine, The Affiliated Hospital of Xuzhou Medical University, Xuzhou, People’s Republic of China

**Keywords:** *Clonorchis sinensis*, IL-33, Type 2 cytokines, Biliary injury, Biliary fibrosis

## Abstract

**Background:**

Clonorchiasis caused by *Clonorchis sinensis* is a zoonotic parasitic disease characterized by cholangitis, biliary proliferation, biliary fibrosis, and even cholangiocarcinoma. Our previous study showed that the expression of interleukin (IL)-33 is increased in both humans and mice infected by *C. sinensis*, suggesting that IL-33 is potentially involved in the pathogenesis of clonorchiasis. However, the roles and potential mechanism of IL-33 underlying remain unknown.

**Methods:**

Wild-type (WT) and *IL-33* knockout (KO) mice (BALB/c female mice) were orally infected with 45 metacercariae of *C. sinensis* for 8 weeks. Biliary injuries and fibrosis were extensively evaluated. Hepatic type II cytokines (IL-4, IL-13, and IL-10) were detected by ELISA.

**Results:**

For wild-type mice, we found that the mice infected with *C. sinensis* showed severe biliary injuries and fibrosis compared with the normal mice that were free from worm infection. In addition, the levels of type II cytokines such as IL-4, IL-13, and IL-10 in infected wild-type mice were significantly higher than in the control mice without infection (*P* < 0.05). However, IL-33 deficiency (*IL-33* KO) prevents the augmentation of biliary injuries and fibrosis caused by *C. sinensis* infection. Furthermore, the increased levels of these type II cytokines induced by worm infection were also reversed in *IL-33* KO mice.

**Conclusion:**

Our present study demonstrates that IL-33 contributes to the pathogenesis of *C. sinensis*-induced biliary injuries and repair, which can potentially orchestrate type 2 responses. These findings highlight the pathophysiological role of IL-33 in the progression of clonorchiasis.

**Supplementary Information:**

The online version contains supplementary material available at 10.1186/s13071-022-05490-6.

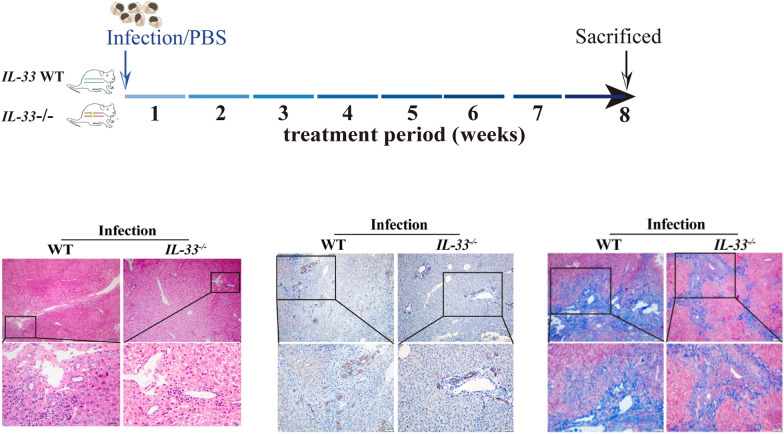

## Background

*Clonorchis sinensis* is a kind of worm that can dwell in the bile ducts for almost 20–30 years, which can cause recurrent pyogenic cholangitis characterized by inflammatory strictures, and can progress to biliary stasis, supportive cholangitis, biliary cirrhosis, and even cholangiocarcinoma [1]. Like other helminths, infection with *C. sinensis* can predominantly trigger type II immune responses, including innate lymphoid cells (ILC) 2, Th2, macrophage (M) 2, and its relative cytokines (IL-4, IL-13, IL-5), which may cause restriction to hyperinflammatory responses and facilitate biliary repairment, although the mechanism remains obscure [2, 3].

IL-33 is a pleiotropic cytokine that can elicit both type I and type II immune responses in a context-dependent manner. During helminth infection, IL-33 is a potent cytokine for expanding ILC2/M2/Tregs and inducing IL-13 production, suggesting that IL-33 is critical to eliciting type 2 immune responses [4, 5]. However, as an ‘alarmin’ for sensing the danger signals, IL-33 seems to play a contradictory role in different stimuli that cause tissue damage [6]. Our previous study showed that the expression of IL-33 was increased in both humans and mice infected with *C. sinensis*, suggesting a potential role of IL-33 in *C. sinensis* infection [7]. Furthermore, other studies also suggest that IL-33 may promote biliary proliferation and repair via IL-33/ILC2/IL-13 circuit and further promote cholangiocarcinogenesis from peribiliary glands, which suggests that IL-33 may play some roles in *C. sinensis*-caused biliary injuries [8]. Given the background, we hypothesize that IL-33-orchestrated type 2 immune responses may cause more severe biliary injuries mediated by *C. sinensis*. To verify our hypothesis, we employed *IL-33* KO and wild-type mice that were administered *C. sinensis* metacercaria for 8 weeks to examine the biliary injuries/fibrosis and immune responses in these mice. The data showed that IL-33-mediated type II immune responses (such as increased production of IL-4, IL-13, and IL-10) exacerbate worm-induced biliary injuries. The data indicate a mechanism underlying IL-33-mediated biliary injury, which contributes to the pathogenesis of cholangiopathy caused by *C. sinensis*.

## Materials and methods

### Preparation of metacercaria *C. sinensis*

Metacercariae of *C. sinensis* were prepared as described elsewhere [9]. Briefly, *C. sinensis* metacercariae were collected from the intermediate host fish *Pseudorasbora Parva* purchased from Guangxi Zhuang Autonomous Region, China. The fish was digested with pepsin-HCl (0.6%) artificial gastric juice. After 12 h, the preparation was filtered through a series of sieves to remove big particles. The metacercariae were collected after sedimentation using a microscope and stored in the refrigerator at 4 °C in phosphate-buffered solution (PBS).

### Mouse model

Wild-type mice and *IL-33* KO mice (BALB/c female, 6–8 weeks, weight 18–20 g) were used for the study. Wild-type mice were purchased from the Beijing Vital River Laboratory Animal Technology Co., Ltd. *IL-33* KO mice were generated using CRISPR/Cas9 technology as previously published [10]. All the mice were bred and housed under specific pathogen-free conditions maintained at 23 °C ± 2 °C with 12 h light/12 h dark cycles at the animal center of Xuzhou Medical University.

Wild-type and *IL-33* KO mice were divided into two groups (*n* = 6 in each group): the normal control group and worm infection group. For worm infection, 45 metacercariae were intragastrically administrated to each mouse, and the irrigating solution was observed under the microscope to ensure that all the metacercariae were completely infused; the mice of the non-infected received the same volume of normal saline. At 8 weeks post-infection, the mice were administrated with 2% pentobarbital under deep anesthesia and killed, and the liver and serum from each mouse were collected for analysis of pathological conditions.

### Serum biochemical analyses

To analyze liver function, the activities of alanine aminotransferase (ALT), aspartate aminotransferase (AST), direct bilirubin (DBIL), and total bile acid (TBA) in the serum were detected using a biochemical analyzer (Cobas 601 analyze System, Roche, Germany) at Department of Laboratory Medicine, Affiliated Hospital of Xuzhou Medical University, China.

### Hematoxylin and eosin (H&E) staining

For histological analysis, partial liver tissue (20 mm × 20 mm × 3 mm) was excised and immersed in 4% paraformaldehyde for 48 h. The tissue was then embedded in paraffin, sliced to a thickness of 4 μm, and routinely stained with H&E according to the manufacturer’s instructions (Jiangsu Beyotime biotechnology research institute, China). The pathological changes of stained histological sections were analyzed by mHAI (modified Histology Activity Index) scores system using a microscope (Olympus, Japan) [11].

### Hydroxyproline contents detection

Hepatic hydroxyproline content was determined using a commercially available kit (Jiancheng Institute of Biotechnology, Nanjing, China) according to the manufacturer's recommendations.

### Masson’s staining

As for Masson’s staining, the section of the liver was embedded in paraffin, sliced to a thickness of 4 μm, and routinely stained with Masson’s trichrome according to the manufacturer’s instructions (Jiancheng, Nanjing, Jiangsu, China) (see Additional file 1). Five lower-power visual fields (× 100 magnifications, Olympus, Japan) were randomly selected from the stained sections of each mouse, and Image J software (NIH, Bethesda, MD, USA) was used to calculate the positive expression of fibrous tissue.

### Immunohistochemistry (IHC) staining

The immunohistological analysis of the liver tissue was performed using 4-μm serial thick sections of embedded tissue from each mouse. Briefly, the liver tissue was deparaffinized, hydrated, and heated in citric acid buffer at 95 °C for 10 min and then blocked with 5% bovine serum albumin (BSA) for 30 min. The slides were then incubated overnight with primary anti-cytokeratin 19 (CK19) (1:500, ab52625, Abcam, Cambridge, USA) or alpha-smooth muscle actin (α-SMA) (1:400, ab124964, Abcam, Cambridge, USA). After the incubation, the slides were washed with PBS, and DAB (1:200, ZSGB-BIO, Beijing, China) was added as an enzyme substrate. Five lower power fields (× 100 magnifications, Olympus, Japan) were randomly selected from each mouse staining section. CK19, α-SMA semi-quantification of signal intensity was done using Image J software (NIH, Bethesda, MD, USA) [12].

### ELISA

The concentration of cytokines in the liver was detected using Enzyme-linked Immunosorbent Assay (ELISA). In each group, the mouse liver homogenate from each mouse was immediately collected to evaluate the concentration of IL-4, IL-10, and IL-13 by a commercial ELISA Kit with 96-well plates (Thermo Scientific, CA, USA). All procedures were performed according to the instructions provided by the kit. Concentrations of cytokine in the serum were calculated using standard curves as references.

### Statistical analysis

All quantitative data were shown as means ± SEM. Differences among more than two groups were assessed by one- or two-way analysis of variance (ANOVA) or Kruskall-Wallis tests for non-parametric data if appropriate. All statistical graphs were drawn using the GraphPad Prism 8.0 statistical package (San Diego, CA). SPSS 23.0 (SPSS Inc, Chicago, IL, USA) was used to perform the statistical analysis. Differences were considered if a *P*-value was < 0.05.

## Results

### Histopathological changes in the liver of IL-33 WT and IL-33 KO mice with *C. sinensis* infection

**Fig. 1:**
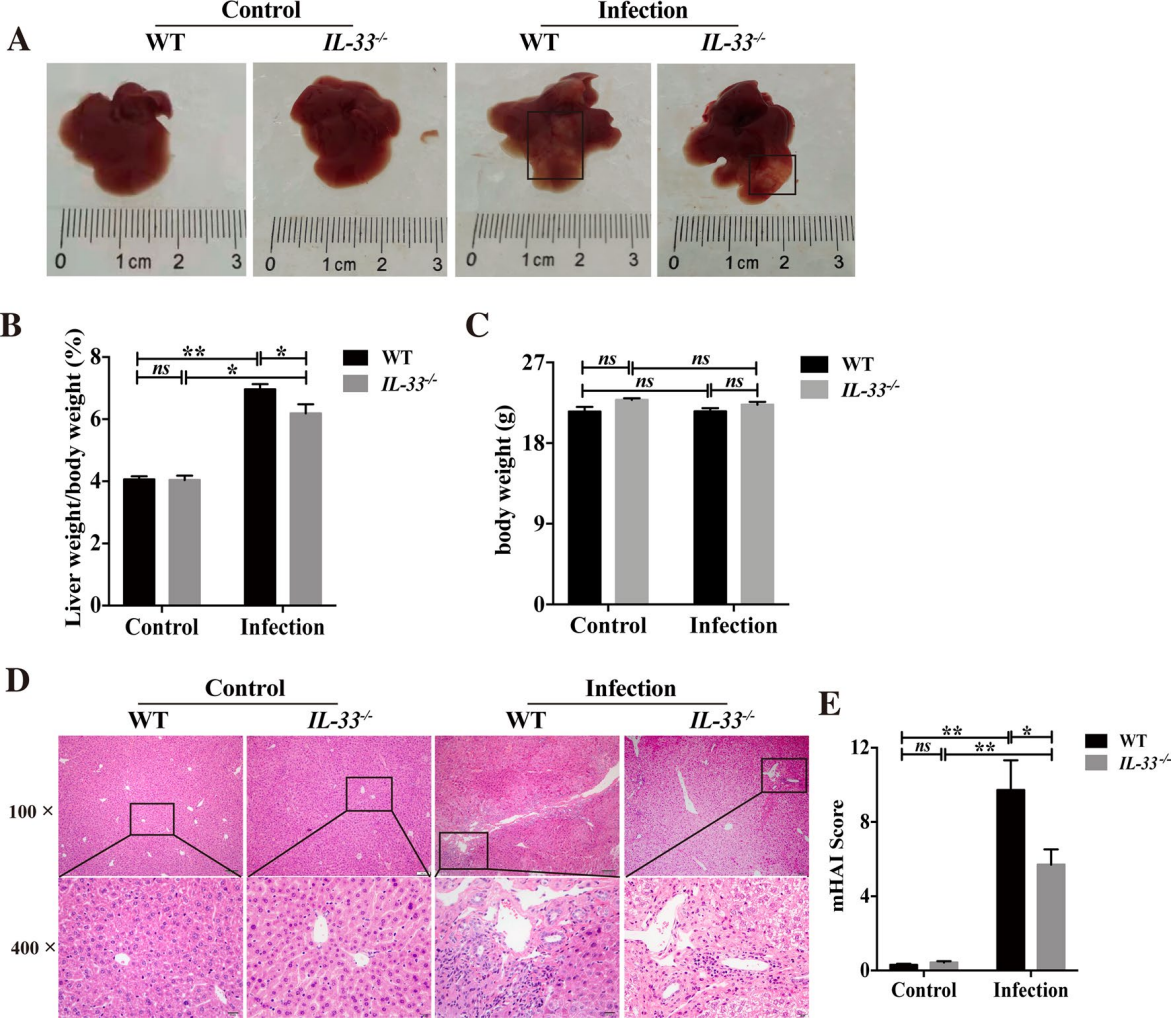
Histopathological observation in the liver of WT and *IL-33* KO mice infected by *Clonorchis sinensis*. BALB/c mice (*n* = 6 mice per group) were orally infected by metacercariae *C. sinensis* or PBS for 8 weeks. **A** Gross changes of the liver. **B** Ratio of liver weight to body weight. **C** Changes of body weight. **D**–**E** Hepatic pathological changes shown by H&E staining. **D** Section of H&E staining was evaluated by mHAI scores (**E**, *n *= 4–5). Compared with indicated groups, *P < 0.05, **P < 0.01. *ns* not significant. All data represent at least three independent experiments

To investigate the role and mechanisms of IL-33 in type 2 immune response during biliary injuries caused by *C. sinensis* infection, we first generated *IL-33* KO mice with the same genetic background (BALB/c) using CRISPR-Cas9 gene-editing systems [10]. Then, we established a biliary injury mouse model by infection with *C. sinensis* for 8 weeks. There are expanded fibrotic nodules on the surface of the liver in the *C. sinensis*-infected WT mice, but the number of fibrotic nodules became less in the *IL-33* KO mice than those in IL-33 WT mice (Fig. 1A). We found that the liver weight/body weight ratio increases significantly in the infected WT group (Fig. 1B, Kruskal-Wallis: *χ*^2^ = 9.380, *df* = 2, *P* = 0.009) compared to the non-infected IL-33 wild-type mice; however, infected *IL-33* KO mice have decreased liver weight/body weight ratio compared with infected IL-33 WT mice (Fig. 1B, Kruskal-Wallis: *χ*^2^ = 6.000, *df* = 1, *P* = 0.014); however, there was no statistical difference in body weight between the groups (Fig. 1C, *P* > 0.05). Hematoxylin and eosin (H&E) staining showed that the infected wild-type mice had severe biliary dysplasia, infiltration of immune cells, and deposition of extracellular matrix (ECM) compared with normal control mice without worm infection, and semi-quantitative analysis by the mHAI score confirmed the observation (Fig. 1D, E, Kruskal-Wallis: *χ*^2^ = 9.271, *df* = 2, *P* = 0.010; see Additional file 1). Compared with WT mice, *IL-33* KO mice showed palliative biliary dysplasia, reduced infiltration of immune cells, and ECM deposition (Fig. 1D, E, Kruskal-Wallis: *χ*^2^ = 4.083, *df* = 1, *P* = 0.043; see Additional file 1).

### IL-33 deficiency prevents biliary injuries in mice infected with *C. sinensis*

**Fig. 2:**
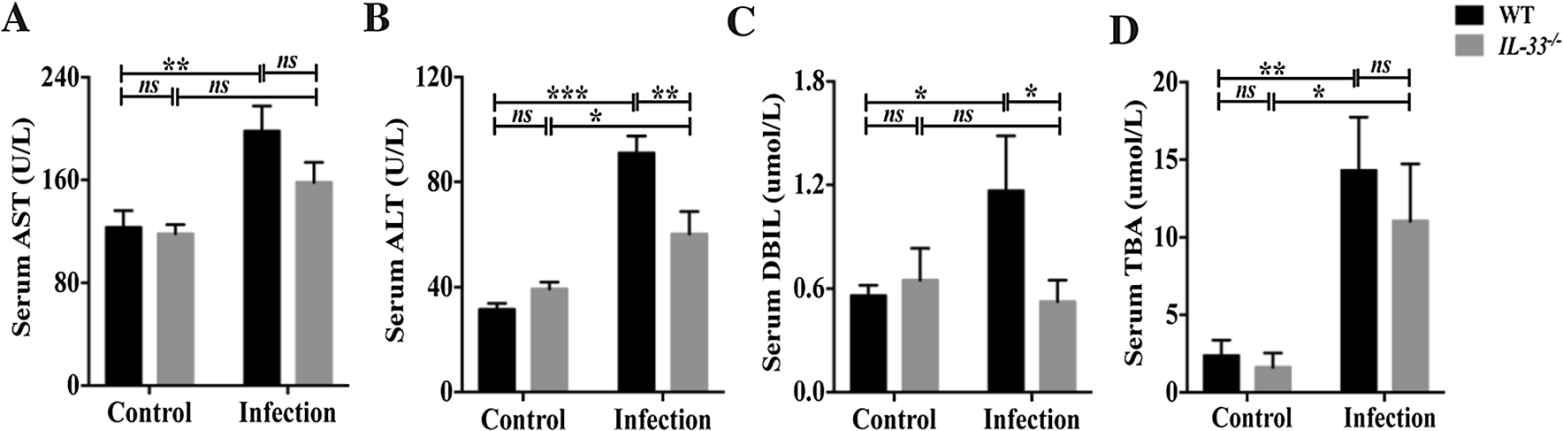
The serum biochemical indicators for hepato-biliary injuries in the liver of WT and *IL-33* KO mice infected by *Clonorchis sinensis*. **A**–**D**: the serum levels of AST **A**, ALT **B**, DBIL **C**, and TBA **D** in these mice (*n* = 3–4). The values were expressed as mean ± SEM. Compared with indicated groups, *P < 0.05, **P < 0.01, ***P < 0.001; *NS* not significant. All data represent at least three independent experiments

We further evaluated serum biochemical indicators of biliary injuries (cholestasis) such as TBA, DBIL, and liver enzymes AST and ALT. We found that the levels of AST [Fig. 2A, ANOVA: *F*_(3, 8)_ = 6.427, *P* = 0.007], ALT [Fig. 2B, ANOVA: *F*_(3, 9)_ = 26.378, *P* < 0.001[, DBIL [Fig. 2C, ANOVA: *F*_(3, 12)_ = 2.762, *P* = 0.026], and TBA [Fig. 2D, ANOVA: *F*_(3, 9)_ = 6.884, *P* = 0.008] were significantly increased after worm infection in wild-type mice, compared with normal control mice, suggesting *C. sinenesis* infection caused severe hepato-biliary injuries. However, the levels of ALT [Fig. 2B, ANOVA: *F*_(3, 9)_ = 26.378, *P* = 0.003] and DBIL [Fig. 2C, ANOVA: *F*_(3, 12)_ = 2.762, *P* = 0.024] were significantly decreased in infected *IL-33* KO mice compared with infected wild-type mice.

**Fig. 3:**
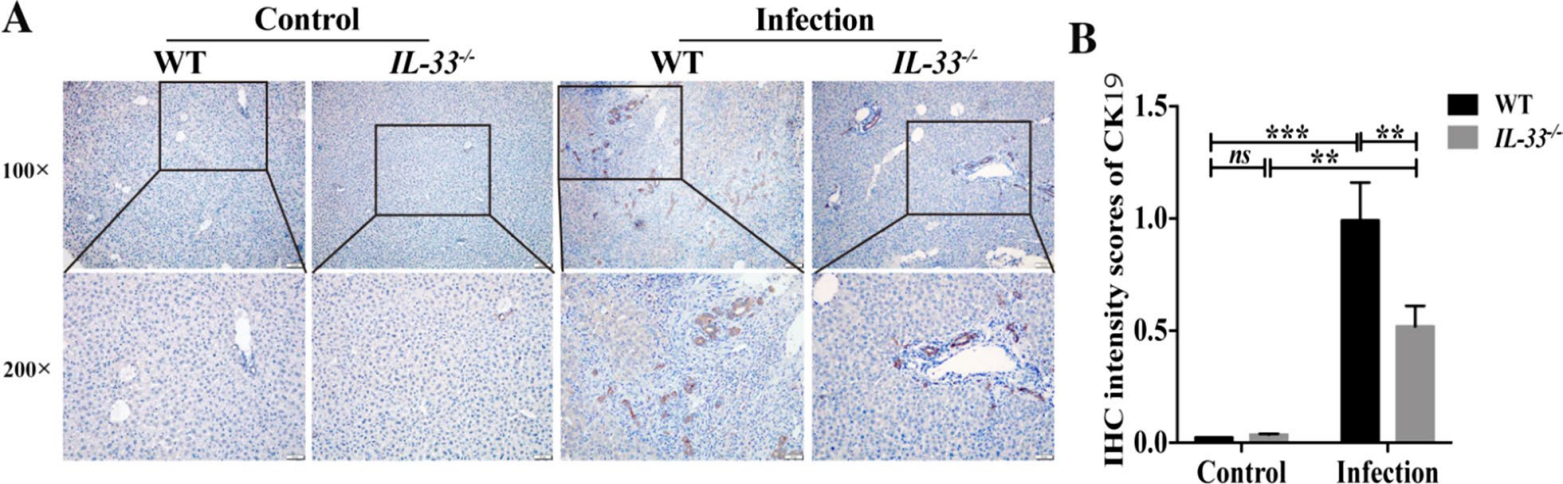
The biliary injuries in the liver of IL-33 WT and IL-33 KO mice infected by *Clonorchis sinensis*. IHC staining of CK19 in all the groups of mice **(A)** and the sections were quantified by integral optical density using Image J software (**B**, *n* = 3–5). The values were expressed as mean ± SEM. Compared with indicated groups, **P < 0.01, ***P < 0.001; *ns* not significant. All data represent at least three independent experiments

In addition, the proliferation of cholangiocytes was evaltuted by IHC staining of CK19, as shown in Fig. 3A, B. *C. sinensis* infection caused more biliary proliferation in infected wild-type mice as shown by the massive proliferation of cholangiocytes compared with the control group [Fig. 3B, ANOVA: *F*_(3, 12)_ = 22.950, *P* < 0.001]; however, the *IL-33* KO mice showed fewer biliary injuries as indicated by IHC staining of CK19 [Fig. 3B, ANOVA: *F*_(3, 12)_ = 22.950, *P* = 0.005; see Additional file 1] compared with these indicators in wild-type mice when they were administrated the same dose of *C. sinensis* metacercaria, suggesting that IL-33 is involved in *C. sinensis*' increased biliary injuries. Collectively, these data suggested that the knocking out of *IL-33* in mice after *C. sinensis* infection decreased biliary injuries.

### IL-33 deficiency reverses biliary fibrosis in *C. sinensis*-infected mice

**Fig. 4:**
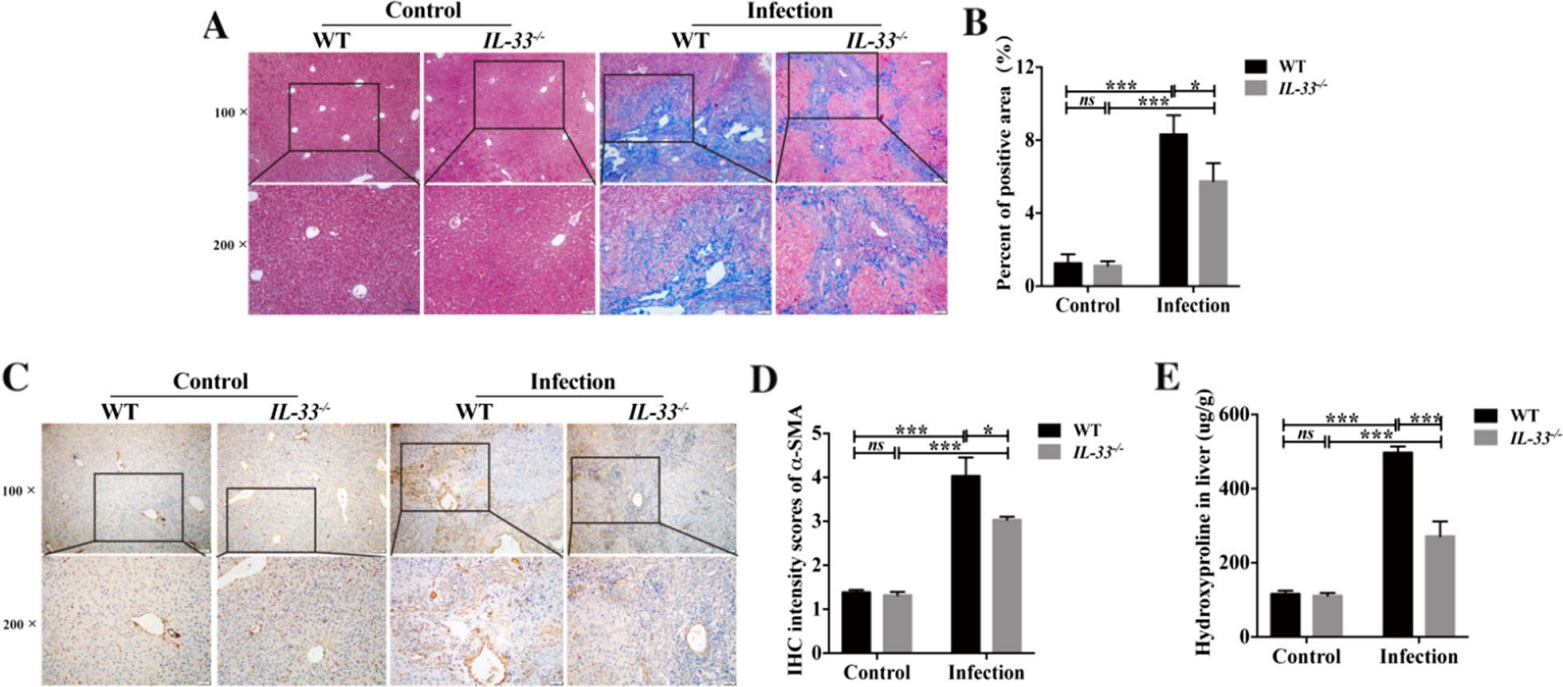
Biliary fibrosis in the IL-33 WT and IL-33 KO mice infected by *Clonorchis sinensis*. **A**–**B** Depositions of extracellular matrix as indicated by Masson’s staining and further semi-quantified by percent of blue area in the hepatic section of mice from all groups (*n* = 4–5). **C**–**D** IHC staining of α-SMA in livers of the mice from all groups; the sections were quantified by integral optical density using Image J software (*n* = 5). (**E**) Hydroxyproline content (*n* = 5). The values were expressed as mean ± SEM. Compared with indicated groups, *P < 0.05, ***P < 0.001; *ns* not significant. All data represent at least three independent experiments

It has previously been demonstrated that IL-33 participates in liver fibrosis regardless of different types of liver fibrosis [8, 13, 14]. Masson’s staining showed extensive blue strips indicating accumulation of ECM in infected wild-type mice compared to control mice [Fig. 4A, B, ANOVA: *F*_(3, 14)_ = 25.302, *P* < 0.001; see Additional file 1]. We also evaluated the expression of α-SMA a marker of activated hepatic stellate cells (HSCs)—the main collagens-producing cells. The levels of α-SMA in the *C. sinensis*-infected wild-type mice were significantly increased compared with non-infected mice [Fig. 4C, D, ANOVA: *F*_(3, 16)_ = 29.319, *P* < 0.001]. In addition, hydroxyproline (Hyp) is the main component of collagen, which can also act as an indicator for fibrosis. In our present study, we found that the wild-type mice infected with *C. sinensis* had a higher level of Hyp compared to normal mice. [Fig. 4E, ANOVA: *F*_(3, 16)_ = 66.114, *P* < 0.001]. In contrast to WT mice infected with *C. sinensis*, we found that IL-33 deficiency caused significant decreases of Hyp contents [ANOVA: *F*_(3, 16)_ = 66.114, *P* < 0.001), α-SMA (ANOVA: *F*_(3, 16)_ = 25.302, *P* = 0.030] and ECM deposition as indicated by Masson’s staining [ANOVA: *F*_(3, 14)_ = 25.302, *P* = 0.028] in *C. sinensis*-*IL-33* KO mice compared with *C. sinensis*-infected wild-type mice, suggesting that IL-33 is critical to the biliary fibrosis caused by *C. sinensis* infection. These data together demonstrated IL-33 deficiency decreases biliary fibrosis during *C. sinensis* infection.

### IL-33 deficiency reduced type 2 cytokines in the liver of *C.-sinensis* infected mice

**Fig. 5:**
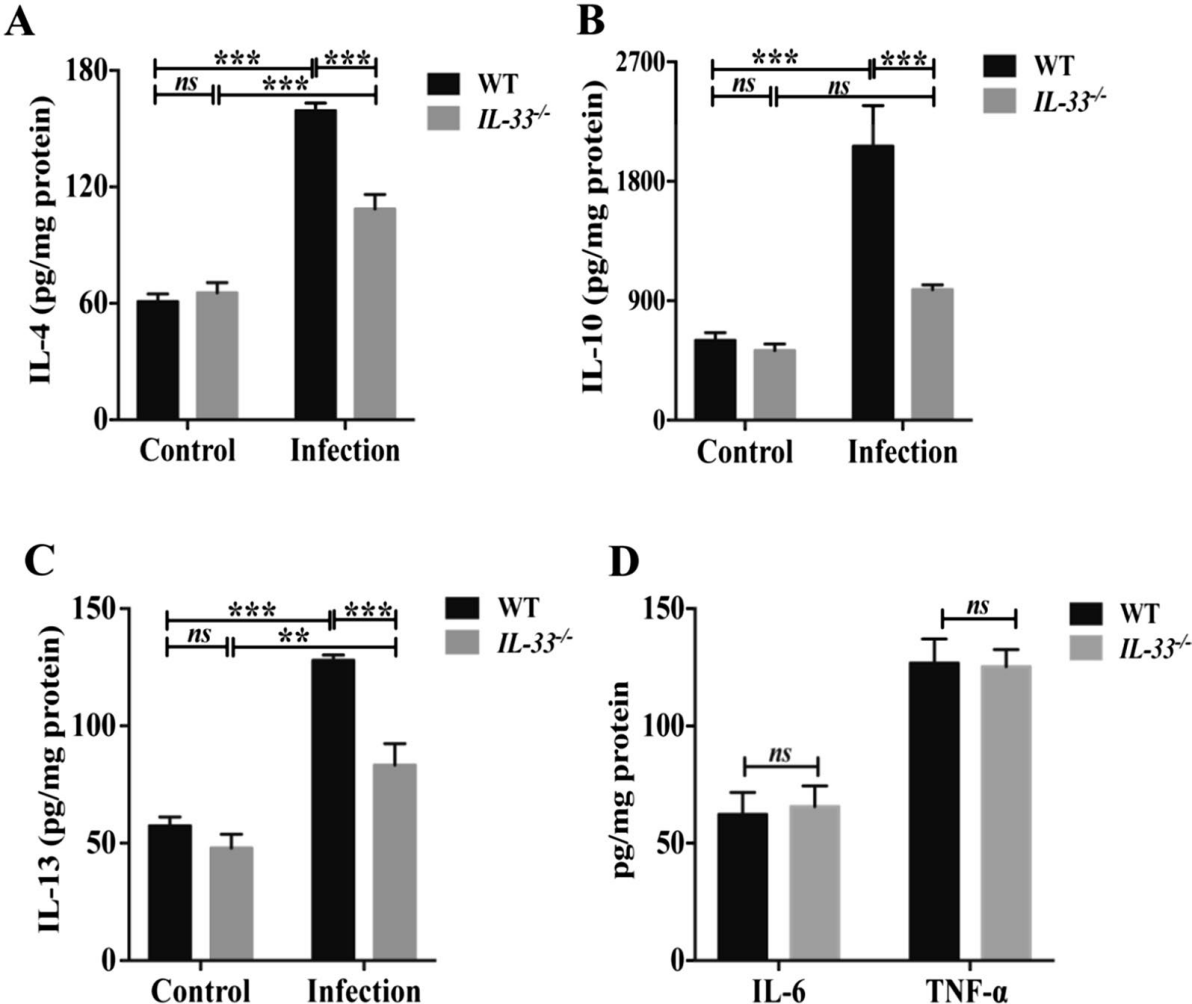
The hepatic type 2 cytokines in WT and *IL-33* KO mice infected by *Clonorchis sinensis*. (**A**–**D**) Levels of IL-4 (**A**), IL-10 (**B**), IL-13 (**C**), IL-6, and TNF-α (**D**) in the liver of mice from all the groups determined by ELISA (*n* = 5–7). The values were expressed as mean ± SEM. Compared with indicated groups, **P < 0.01, ***P < 0.001; *ns* not significant. All data represent at least three independent experiments

Since type II cytokines are critical to biliary dysplasia and repair, which can be orchestrated by IL-33, we further investigated the effects of IL-33 on hepatic levels of IL-4, IL-10, and IL-13 in these groups of mice. In agreement with the previous study, we found that these type 2 cytokines [Fig. 5A, ANOVA: *F*_(3, 20)_ = 66.151, *P* < 0.001; Fig. 5B, ANOVA: *F*_(3, 20)_ = 15.330, *P* < 0.001; Fig. 5C, ANOVA: *F*_(3, 18)_ = 34.248, *P* < 0.001] were significantly increased in wild type after worm infection compared with the non-infection group (normal control mice). However, *IL-33* KO decreased the concentration of IL-4 [Fig. 5A, ANOVA: *F*_(3, 20)_ = 66.151, *P* < 0.001), IL-10 (Fig. 5B, ANOVA: *F*_(3, 20)_ = 15.330, *P* < 0.001)], IL-13 (Fig. 5C, ANOVA: *F*_(3, 18)_ = 34.248, *P* < 0.001] in the liver of *C. sinensis*-infected mice compared with *C. sinensis*-infected *IL-33* wild-type mice. However, type 1 cytokines such as IL-6 and TNF-α (Fig. 5D) were not statistically different between the two groups.

## Discussion

Our previous study showed that IL-33/ST2 participated in the process of clonorchiasis in both human and mouse, but the potential underlying mechanisms remain unclear [7]. In our present study, we investigated the role and mechanisms of IL-33 in the pathogenesis of clonorchiasis using IL-33 wild-type and* IL-33* germ knockout mice with BALB/c background mice. Here, we found that IL-33 contributes to biliary injuries and repair caused by infection with *C. sinensis* via type 2 cytokines. Our present study highlights the pathophysiological roles of IL-33 in the liver in the progress of cholangiopathies. Although not all strains of mice are susceptible to *C. sinensis* infection [15, 16], our and other laboratories have demonstrated that BALB/c mice are relatively susceptible mice which can show cholestasis, recruitment of massive immune cells, severe biliary proliferation, and fibrosis [9, 16–19]. Unfortunately, in our present study, any attempts to find the adult or junior worms failed, maybe because the worms may be expelled although the biliary injuries and fibrosis still existed in BALB/c mice at 8 weeks post-infection [16]. However, it should be further determined whether IL-33 can regulate the development and survivials of worms or not in the following studies. 

It has previously been shown that IL-33 can activate several types of cells involved in type 2 immune response including ILC2s, mast cells, Th2 cells, eosinophils, basophils, and dendritic cells, and alternatively activated macrophages (AAM or M2) [20, 21]. It’s well known that type 2 immune response plays a very important role in wound healing and tissue repair during helminth infection, however, the excessive inflammatory reaction during helminth infection can lead to the excessive accumulation of extracellular matrix and collagen deposits leading to fibrosis [22]. In *C. sinensis* infection, the hyperactivation of AAM is involved in biliary fibrosis by the activation of beta 2 adrenergic receptors [23]. Nevertheless, the mechanisms of IL-33 in type 2 immune response during *C. sinensis* infection are largely unknown. In this study, we found that the infection of mice with *C. sinensis* was associated with an increase in hepatobiliary damage and liver function, while the deletion of *IL-33* gene in mice was associated with a decrease in biliary fibrosis in infected mice. The control of the inflammatory reaction is an important element during liver and biliary injury and fibrosis. Our results revealed that the deletion of IL-33 in mice was associated with a decrease in the production of type 2 cytokines followed by a decrease in biliary injury. The increased expression of IL-33 and type 2 cytokines have been shown to induce the activation of hepatic stellate cells leading to an increase in collagen deposit [24, 25]. This assertion is in agreement with the results of this study. Indeed, we demonstrated that the elevated type 2 cytokines were associated with the increase in biliary fibrosis as shown by Masson staining, Hyp, and α-SMA.

As mentioned above, liver fibrosis is characterized by excess ECM deposition. The elevated expression of IL-33 is associated with the activation of IL-33 receptor (IL-33R) through ST2 signaling, leading to the increased production of IL-4 and IL-13 with the increase in liver fibrosis [26, 27]. In this study, we found that the deletion of IL-33 in mice lead to a significant decrease in biliary fibrosis in infected mice. The infection of mice with *Leishmania donovani* has been shown to induce liver damage by the suppression of type 1 immune response through IL-33/ST2 axis [28]. The activation of IL-33/ST2 signaling has also been shown to be positively correlated with the severity of primary biliary cholangitis [29]. All these data together suggest that the activation of IL-33 could be associated with a poor prognosis in liver fibrosis by the upregulation of type 2 immune response, leading to the increase in type 2 cytokines which are mainly the cause of biliary injuries and fibrosis. The benefits of the inhibition or the deletion of IL-33 have been shown in different types of conditions involving type 2 immune response, which suggests that IL-33 may be the subject of more investigation in the future potential therapeutic target of biliary injuries and fibrosis caused by *C. sinensis*.

## Conclusion

In summary, our present study investigated the roles and mechanisms of IL-33 biliary injuries and fibrosis caused by *C. sinensis* infection. We found that IL-33 advanced biliary injuries and fibrosis in mice caused by *C. sinensis* since the biliary injuries and repairment in our mouse model were retained due to the deficiency of IL-33 in *IL-33* KO mice. Therefore, we highlight the importance of the IL-33 in the pathogenesis of cholangiopathies caused by *C. sinensis*. Further studies are warranted to discover the source and pathogenic mechanism of these cytokines in our model.

## Supplementary Information

**Additional file 1.** The quantitative details and statistics of histological analysis in the present study.**Table 1** Statistics of H&E staining;**Table 2** Statistics of Masson’s staining;**Table 3** Statistics of IHC staining for CK19; **Table 4** Statistics of IHC staining forα-SMA.

## Data Availability

The authors confirm that the data supporting the findings of this study are available within the manuscript and its supplementary data.

## Abbreviations

AAM or M2: Alternatively activated macrophages
ANOVA: One-way analysis of variance
ALT: Alanine aminotransferase
AST: Aspartate aminotransferase
α-SMA: Alpha smooth muscle actin
BSA: Bovine serum albumin
CK19: Cytokeratin 19
*C. sinensis*: *Clonorchis sinensis*
DBIL: Direct bilirubin
ECM: Extracellular matrix
ELISA: Enzyme-linked immunosorbent assay
HSCs: Hepatic stellate cells
Hyp: Hydroxyproline
IHC: Immunohistochemistry
IL: Interleukin
ILC: Innate lymphoid cells
KO: Knockout
M: Macrophage
PBS: Phosphate-buffered solution
TBA: Total bile acid
WT: Wild type
