# Supplementary material for: Interleukin-33 deficiency prevents biliary injuries and repairments caused by Clonorchis sinensis via restraining type 2 cytokines
Source: Parasit Vectors. 2022 Oct 22;15:386. doi: 10.1186/s13071-022-05490-6 (PMC9587592; doi:10.1186/s13071-022-05490-6)
Supplement: Supplementary file 1 — Additional file 1. The quantitative details and statistics of histological analysis in the present study.Table 1 Statistics of H&E staining;Table 2 Statistics of Masson’s staining;Table 3 Statistics of IHC staining for CK19; Table 4 Statistics of IHC staining forα-SMA. [file 13071_2022_5490_MOESM1_ESM.docx]

**The quantitative details of histological analysis:**

1. **H&E staining:**

According to the reference (Goodman ZD. Grading and staging systems for inflammation and fibrosis in chronic liver diseases[J]. *J Hepatol*, 2007. 47(4):598-607.), five fields of view (× 100) of each section (per mouse) were selected for histopathological scoring, and the average value of these five pictures was taken as the score of this mouse for the following statistical analysis.

1. **Masson’s staining and Immunohistochemistry (IHC) staining:**

Five fields of view (× 100) were selected randomly to represent the one section (each mouse liver) for histological analysis, every picture was processed by imageJ to obtain the percentage of positive area in the total tissue area, and the average value of these five pictures was taken as the histological score of this section for the following atatistical analysis.

**Statistics:**

**Table 1 H&E staining:**

|  | **WT** | | | | | ***IL-33^-/-^*** | | | | |
| --- | --- | --- | --- | --- | --- | --- | --- | --- | --- | --- |
| **Control** | 0.333 | 0.167 | 0.167 | 0.333 | 0.500 | 0.667 | 0.333 | 0.333 | 0.333 | 0.500 |
| **Infection** | 7.667 | 8.400 | 8.333 | 14.500 |  | 4.167 | 8.000 | 5.167 | 5.500 |  |

1. **Table 2 Masson’s staining:**

|  | **WT** | | | | | ***IL-33^-/-^*** | | | | |
| --- | --- | --- | --- | --- | --- | --- | --- | --- | --- | --- |
| **Control** | 1.169 | 0.986 | 0.369 | 0.673 | 3.106 | 0.962 | 1.252 | 0.716 | 0.682 | 2.014 |
| **Infection** | 7.217 | 8.239 | 11.24 | 6.489 |  | 2.977 | 7.241 | 5.801 | 7.016 |  |

1. **Table 3 IHC staining of CK-19:**

|  | **WT** | | | | | ***IL-33^-/-^*** | | | | |
| --- | --- | --- | --- | --- | --- | --- | --- | --- | --- | --- |
| **Control** | 0.025 | 0.026 | 0.020 | 0.023 |  | 0.035 | 0.030 | 0.044 |  |  |
| **Infection** | 0.705 | 1.482 | 0.926 | 0.853 |  | 0.670 | 0.293 | 0.665 | 0.450 |  |

**4Table 4 IHC staining of α-SMA:**

|  | **WT** | | | | | ***IL-33^-/-^*** | | | | |
| --- | --- | --- | --- | --- | --- | --- | --- | --- | --- | --- |
| **Control** | 1.249 | 1.502 | 1.406 | 1.276 | 1.493 | 1.050 | 1.408 | 1.503 | 1.349 | 1.277 |
| **Infection** | 3.452 | 3.078 | 5.896 | 3.856 | 4.493 | 3.084 | 2.993 | 3.248 | 3.026 | 2.830 |
